# Supplementary material for: The petal identity gene PhDEF has a major binding and regulatory action in the epidermis
Source: eLife. 2026 Oct 15;15:RP113154. doi: 10.7554/eLife.113154 (PMC13671715; doi:10.7554/eLife.113154)
Supplement: Peer review history

# Editor report: eLife Assessment

**DOI:** 10.7554/eLife.113154.2.sa0

**Authors:** Detlef Weigel

**Affiliations:** Max Planck Institute for Biology Tübingen Germany

**Keywords:** Solid, Valuable

This **valuable** study investigates the action of a floral homeotic transcription factor in different cell types. The results provide **solid** evidence that PhDEF shows more extensive binding and regulatory effects in the epidermis than in the mesophyll, supporting the conclusion that cell-layer context influences homeotic gene function. While there are some limitations, they do not undermine the principal observation of layer-dependent PhDEF activity, but the interpretation of the data is complicated by uncertainties in cell-type assignment and composition in the scRNA-seq data, the cell-type-enriched rather than strictly cell-type-specific nature of the ChIP-seq data, and differences in developmental stage between the single-cell and ChIP-seq experiments. The work will be of interest to developmental biologists studying transcriptional regulation and cell identity.

[Editors' note: this paper was reviewed by Review Commons.]

# Referee report: Reviewer #1 (Public review)

**DOI:** 10.7554/eLife.113154.2.sa1

Summary:

The authors previously generated two cell-layer-specific mutants of petunia for the petal identity gene PhDEF. In this study, they profiled differential gene expression in those mutants through single-cell RNA sequencing (scRNA-seq). They found that more genes are highly and specifically expressed in the epidermal cell layer than in mesophyll cells. In addition, they identified cell-layer-specific and -aspecific PhDEF target genes. Using the extensive single-cell transcriptome and layer-specific target identification, the authors concluded that different cell identities affect homeotic regulator PhDEF, thereby influencing transcriptional regulation.

Major comments from first round of review:

This presented work provides comprehensive evidence, that pre-existing cell layer identity (epidermis and mesophyll) modulate transcriptional output of homeotic transcription factor, PhDEF.

However, a disconnection between PhDEF bindings to genome and transcriptional output undermines the robustness of their conclusion although some binding loci were shown to be correlated with DEG. This may indicate the chromatin state, the existence of interacting partners, and the non-productive binding of PhDEF, suggesting that PhDEF binding alone is not sufficient to predict transcriptional outcomes and additional regulatory mechanisms that shape gene expression in addition to the layer-specific regulatory mechanisms. This disconnection may also be due to the developmental timing. Indeed, it appears authors used different flower stages for ChIP-seq and scRNA-sequencing. In fully differentiated organs, PhDEF binding itself may be no longer transcriptionally productive, and differential gene expression results primarily from the pre-established cell identity rather than directly from the homeotic regulation of PhDEF. Therefore, the main question the authors asked-how homeotic identity works with cell-layer identity and how the homeotic gene, PhDEF, acts in mature organs-was not clearly explained by this study.

In Figure 2, the use of the term "target" is potentially misleading. It sounds like direct target genes (direct binding and differential expression) for PhDEF, but it refers only to DEGs.

Lines 496-497: When the authors state, "~ demonstrates for the first time that the regulatory function of homeotic factor is influenced by cell layer identity," it sounds overstated, as prior studies have shown that pre-existing tissue or cell identity can shape transcriptional activity and developmental output.

Significance:

This study is well-designed and technically sound. They utilize single-cell transcriptomics and ChIP-seq by using genetically well-defined genetic materials and layer-specific PhDEF deletion mutants. The analysis showed where PhDEF binds to genomic loci and which genes are differentially expressed in petal epidermis and mesophyll, providing evidence of cell-layer-specific function of homeotic gene in mature organs. Although certain mechanistic aspects were not elucidated, the data from the extensive genome-wide study contributed to drawing their conclusions.

Advances: This research goes beyond classical models of floral organ identity by showing that homeotic gene function is not uniform in the same floral organ. It represents a conceptual advance in our understanding by integrating cell layer identity into the framework of homeotic gene regulation.

Audience: This study will be of broad interest to scientists who study transcription networks, cell and organ identity in the context of plant development.

My field of expertise: Transcriptional regulation by transcription factor, epigenetic regulation of gene expression, plant development.

Comments on latest version:

Thank you for sharing the assessment. I am happy with the proposed eLife assessment. I do not have any further amendments to my review.

# Referee report: Reviewer #2 (Public review)

**DOI:** 10.7554/eLife.113154.2.sa2

Summary:

This study from Cavallini-Speisser et al. cleverly leverages a tissue layer-specific mutant, single cell and bulk RNA-sequencing, and ChIP-sequencing to decipher tissue layer-specific regulation of petal development in petunia by the PhDEF transcription factor. The authors find common and unique targets of PhDEF between the epidermis and mesophyll and conclude that the activity of transcription factors like PhDEF are influenced by the pre-existing environment in the cell they are expressed in. Understanding when and how a given transcription factor drives expression of unique target genes in various contexts is an important aspect of developmental biology that can be elusive outside of highly tractable model systems. As such, I think this study is of high value and has strong potential to expand our understanding of how developmental specificity is mediated by commonly employed transcriptional regulators. However, I think there are some issues with possible over-interpretation and some places where documentation of experimental design and data quality control are lacking. I elaborate on these concerns below.

Major Comments From Review at Review Commons:

(1) Line 155: Assigning mesophyll clusters by default without any positive marker genes strikes me as problematic, especially as much of the analysis rests on comparing the transcriptomes of epidermis and mesophyll cells. Can the authors perhaps leverage published scRNA-seq datasets to find potential mesophyll markers, even homologs from other species, to improve confidence in the cluster assignment?

(3) Line 228: Through the description and interpretation of the ChIP-seq dataset, the authors use the fact that peaks are more abundant and bigger in the epidermis to conclude that binding of PhDEF is "stronger" in the epidermis. This implies a difference in physical interaction between the TF and the DNA that I don't think can be concluded from the data presented. This could be confounded by biology; if expression of PhDEF is more heterogeneous in the mesophyll than in the epidermis, the peaks from that tissue will be averaged out and appear smaller when in fact the binding is the same strength. This could also be a technical artifact if the ChIP was less efficient in one sample versus another. This conclusion requires reinterpretation. The authors have the power to address this at least partially with the scRNA-seq by measuring PhDEF heterogeneity. I believe assessing ChIP efficiency would have required a spike in control, but perhaps there is a computational way to address this. It is important to discuss these confounding factors in the text.

(3) Line 376: The authors risk overinterpreting a lack of differential gene expression detection in their analysis of PhDEF binding profiles. This can be affected by how deeply a library was sequenced or how many cells were analyzed per cell type. A gene might not be found to be DE if low depth or few cells resulted in noise or dropout. Lack of detection does not mean lack of differential regulation so the biological relevance of this portion of the analysis should be interpreted with caution.

(4) Line 476: The authors state there is a mismatch in developmental timing between the RNAseq and ChIP datasets. Why is this? This is mentioned briefly in the Discussion, but has the potential to be majorly confounding to the joint interpretation of the ChIP and RNAseq datasets. This experimental design choice should be justified more thoroughly and a consideration of the limitations it brings to data interpretation should be more prominent in the text.

Significance:

Strengths: The authors employ a unique and powerful mutant system to explore a fundamental developmental biology question. In addition, the datasets generated will likely be useful to other researchers working in petunia or flower development.

Limitations: While the mutant system employed here is a creative way to get at tissue-layer specific transcription factor activity, the ChIP samples still include heterogeneous cell types, which may impact the findings presented here.

Advance: This study uses a unique system to test the function of a transcription factor in distinct cell types. As stated above, understanding when and how a given transcription factor drives expression of unique target genes in various contexts is an important aspect of developmental biology.

Audience: I believe this work will be of primary interest to the plant development, single cell, and chromatin biology communities. These are specialized, basic research communities.

Reviewer Expertise: I am a plant developmental biologist who works with multiple modes of cell-type-specific NGS datasets including bulk and single cell RNAseq and ChIPseq among others.

Comments on latest version:

I find the authors' response to my review to be comprehensive and I think the revision looks solid. The eLife assessment works for me.

# Referee report: Reviewer #3 (Public review)

**DOI:** 10.7554/eLife.113154.2.sa3

Summary:

This study addresses a fundamental but underexplored aspect of homeotic gene function: how regulators of cell identity act during late stages of organ development. The authors take advantage of layer-specific mutants of the MADS-box gene PhDEF in Petunia hybrida to dissect the roles of this floral identity regulator in the epidermis and mesophyll. By combining single-cell RNA sequencing with ChIP-seq analyses in wild-type and mutant chimeric petals, the work demonstrates that, although PhDEF is expressed at comparable levels in both petal layers, it binds to and regulates a substantially larger and more layer-enriched set of genes in the epidermis than in the mesophyll. The identification of both layer-specific and shared PhDEF binding sites supports a model in which pre-existing layer identity modulates the regulatory output of homeotic transcription factors.

Major comments from review at Review Commons:

Dissecting PhDEF binding preferences in the epidermis versus the mesophyll using the star and wico mutants is a clever and powerful approach. However, conclusions involving cell identity should be drawn with caution for two reasons. First, cell identities appear to be altered in the mutants: scRNA-seq data suggest that even cells assigned to the same cluster can be molecularly distinct across genotypes. For example, the transcriptomes of wild-type epidermis and mesophyll are highly similar (Pearson correlation R = 0.94), yet both show lower correlation with epidermal cells from star or wico petals. These results raise questions such as are the cells identified as epidermal cells really strictly epidermal in the mutants? Do you need to take into consideration cell composition of the mutants when you do differential expression analysis? Second, PhDEF is expressed in both epidermal and mesophyll clusters in all genotypes, albeit at different levels and in fewer cells in the mutants. As a result, the ChIP-seq profiles should be interpreted as cell type-enriched rather than cell type-specific. And there are a few things that need clarification:

a) Protoplasting and tissue dissection analyses suggest that mesophyll cells constitute more than 80% of the cells in wild-type petals, whereas the scRNA-seq data indicate a substantially lower proportion. Could this discrepancy reflect technical biases in cell recovery or capture efficiency, or issues related to cell identity assignment during clustering and annotation? Notably, the scRNA-seq data from star and wico petals show mesophyll proportions close to 80%. Is this difference due to an increased abundance of mesophyll cells in the mutants, or could it instead reflect differences in transcriptomic separability? In wild-type petals, the epidermal and mesophyll transcriptomes are highly correlated and express similar numbers of genes, with epidermal cells distinguished mainly by higher expression of a subset of genes. This raises the possibility that mesophyll cells in the wild type occupy a more plastic or less differentiated transcriptional state and may therefore be misclassified as epidermal cells, whereas disruption of regulatory mechanisms in the mutants enhances transcriptional divergence and alters cell clustering outcomes.

b) Cluster 0 appears to show internal heterogeneity, as the expression patterns of KCS3 and LLE2 are largely mutually exclusive. Do these patterns reflect the presence of distinct epidermal cell types within the limb that are currently grouped into a single cluster?

c) Clusters 0 and 7 both exhibit high expression of pigmentation genes, while cluster 7 additionally shows strong enrichment for cell division genes. Are cell cycle genes the primary features distinguishing these two clusters? If cell cycle effects are regressed out, would cluster 7 merge with cluster 0, potentially yielding a more continuous cell state trajectory and helping to resolve the pattern noted in point (b)?

d) Pearson correlation is largely driven by highly expressed genes and may therefore be insensitive to changes in cell identity markers. The conclusions that the less clear separation of mesophyll and epidermal cells in star is due to altered cell identity would be more convincing if supported by independent validation, such as in situ hybridization or reporter analyses, to directly visualize molecular alterations in the relevant cell types when comparing wild-type and mutant tissues.

Significance:

Overall, this work significantly advances our understanding of late homeotic gene function. It establishes compelling evidence for how developmental context constrains transcription factor activity and offers broadly relevant insights for studies of organ patterning. The combination of genetic mosaics with single-cell and chromatin-level analyses represents a powerful and generalizable strategy that will be of interest to both plant developmental biologists and researchers studying transcriptional regulation.

My expertise: single cell genomics, chromatin biology and plant development.

Comments on latest version:

The authors have addressed my concerns and questions. And I agree with your assessment of "valuable" for significance and "solid" for strength of evidence.

# Author comment: Author response

**DOI:** 10.7554/eLife.113154.2.sa4

**Authors:** Emma Desert, Quentin Cavallini-Speisser, Evelyne Duvernois-Berthet, Pierre Chambrier, Patrice Morel, Brice Letcher, Carine Rey, Jeremy Just, Suzanne Rodrigues Bento, Daniel Bouyer, Marie Monniaux

**Affiliations:** Laboratoire Reproduction et Développement des Plantes Lyon France; Laboratoire Reproduction et Développement des Plantes Lyon France; Laboratoire Reproduction et Développement des Plantes Lyon France; Laboratoire Reproduction et Développement des Plantes Lyon France; Laboratoire Reproduction et Développement des Plantes Lyon France; Laboratoire de Biologie et Modélisation de la Cellule Lyon France; Centre International de Recherche en Infectiologie Lyon France; Laboratoire Reproduction et Développement des Plantes Lyon France; Laboratoire Reproduction et Développement des Plantes Lyon France; Laboratoire Reproduction et Développement des Plantes Lyon France; Laboratoire Reproduction et Développement des Plantes Lyon France

**General Statements**

We wish to bring to the attention of the editor and reviewers that the title of the manuscript has been modified to more accurately reflect the conclusions of our study, i.e. that PhDEF has a major binding and regulatory role in the petal epidermis.

**Point-by-point description of the revisions**

We thank the three reviewers for their critical reading of our manuscript. We also appreciate that the three reviewers highlighted the conceptual advances and the broad findings that our study brings to the community. They have identified key limitations of our analyses, and we have either provided explanations for our choice, or performed new analyses to circumvent biases. We believe that the manuscript is greatly improved and that our main conclusion, that PhDEF has a major binding and regulatory action in the petal epidermis, is strongly supported by our data.

**Reviewer #1 (Evidence, reproducibility and clarity):**

Summary:

The authors previously generated two cell-layer-specific mutants of petunia for the petal identity gene PhDEF. In this study, they profiled differential gene expression in those mutants through single-cell RNA sequencing (scRNA-seq). They found that more genes are highly and specifically expressed in the epidermal cell layer than in mesophyll cells. In addition, they identified cell-layer-specific and -aspecific PhDEF target genes. Using the extensive single-cell transcriptome and layer-specific target identification, the authors concluded that different cell identities affect homeotic regulator PhDEF, thereby influencing transcriptional regulation.

Major comments:

This presented work provides comprehensive evidence, that pre-existing cell layer identity (epidermis and mesophyll) modulate transcriptional output of homeotic transcription factor, PhDEF.

However, a disconnection between PhDEF bindings to genome and transcriptional output undermines the robustness of their conclusion although some binding loci were shown to be correlated with DEG. This may indicate the chromatin state, the existence of interacting partners, and the non-productive binding of PhDEF, suggesting that PhDEF binding alone is not sufficient to predict transcriptional outcomes and additional regulatory mechanisms that shape gene expression in addition to the layer-specific regulatory mechanisms. This disconnection may also be due to the developmental timing. Indeed, it appears authors used different flower stages for ChIP-seq and scRNA-sequencing. In fully differentiated organs, PhDEF binding itself may be no longer transcriptionally productive, and differential gene expression results primarily from the pre-established cell identity rather than directly from the homeotic regulation of PhDEF. Therefore, the main question the authors asked-how homeotic identity works with cell-layer identity and how the homeotic gene, PhDEF, acts in mature organs-was not clearly explained by this study.

We thank the reviewer for raising this important issue. We agree that the difference in developmental timing between the scRNA-Seq and ChIP-Seq experiments might contribute to the disconnection that this reviewer pointed out.

First, we want to explain that the reason for performing scRNA-Seq on fully differentiated petals was purely technical, as we were initially aiming to obtain protoplasts at stage 8 (stage used for the ChIP-Seq) but could never retrieve enough of them for proper encapsulation in the 10X Chromium chips. We have now clearly explained this in the manuscript (lines 104-107).

A hypergeometric test shows that our ChIP-Seq and scRNA-Seq datasets overlap more than by chance (p = 0.000137); however, we agree that differences in developmental stages possibly bring a confounding effect to our conclusions. Therefore, we have decided to add to our manuscript the intersection between ChIP-Seq and bulk RNA-Seq data performed on WT, star and wico flowers at stage 8, that we published previously (Chopy et al., 2024). In that case, both datasets have been obtained with the same exact genetic material and at the same exact developmental stage.

This intersection confirms that very similar binding profiles are observed for PhDEF target genes, whether they are differentially expressed in the epidermis (star only), in the mesophyll (wico only) or in both layers (star and wico) (Figure 4A). However, star-specific DEGs were more often bound by PhDEF by epidermis+shared binding sites, and wico-specific DEGs more often with mesophyll-specific binding sites, suggesting a weak but significant association between binding and regulatory profiles. Performing similar tests for individual binding categories for DEGs identified by scRNA-Seq also revealed that epidermis-specific DEGs displayed more epidermis+shared binding sites than expected. Since the association between epidermal DEGs and shared+epidermal binding sites is found both in the intersection with RNA-Seq and scRNA-Seq data, we have now stated that “layer-specific binding and transcriptional regulation are partially linked, at least in the epidermis” (line 354).

In Figure 2, the use of the term "target" is potentially misleading. It sounds like direct target genes (direct binding and differential expression) for PhDEF, but it refers only to DEGs.

Indeed, the term "target" was referring to both indirect and direct targets of PhDEF. To avoid any possible confusion, we have replaced it by differentially expressed genes (DEGs) throughout the manuscript, when appropriate.

Lines 496-497: When the authors state, "~ demonstrates for the first time that the regulatory function of homeotic factor is influenced by cell layer identity," it sounds overstated, as prior studies have shown that pre-existing tissue or cell identity can shape transcriptional activity and developmental output.

We agree that previous studies have shown that cell identity influences transcriptional activity in general. While this might not have been specifically assessed in the context of different cell layers, we have rewritten this sentence accordingly.

Minor comments:

In the UMAP presentation, as depicted in Figures 2C, S3, and S5, the cells with zero expression can be colored in light gray (or an inverted color scheme). The purple hue masks the gene expressions of other cells, making it difficult to see the yellow or light green colored cells.

We have modified all UMAPs depicting gene expression as suggested, in Figures 2C and S5, and replaced UMAPs with DotPlots in Figure S3.

**Reviewer #1 (Significance):**

General assessment: This study is well-designed and technically sound. They utilize single-cell transcriptomics and ChIP-seq by using genetically well-defined genetic materials and layer-specific PhDEF deletion mutants. The analysis showed where PhDEF binds to genomic loci and which genes are differentially expressed in petal epidermis and mesophyll, providing evidence of cell-layer-specific function of homeotic gene in mature organs. Although certain mechanistic aspects were not elucidated, the data from the extensive genome-wide study contributed to drawing their conclusions.

Advances: This research goes beyond classical models of floral organ identity by showing that homeotic gene function is not uniform in the same floral organ. It represents a conceptual advance in our understanding by integrating cell layer identity into the framework of homeotic gene regulation.

Audience: This study will be of broad interest to scientists who study transcription networks, cell and organ identity in the context of plant development.

My field of expertise: Transcriptional regulation by transcription factor, epigenetic regulation of gene expression, plant development

**Reviewer #2 (Evidence, reproducibility and clarity):**

Summary:

This study from Cavallini-Speisser et al. cleverly leverages a tissue layer-specific mutant, single cell and bulk RNA-sequencing, and ChIP-sequencing to decipher tissue layer-specific regulation of petal development in petunia by the PhDEF transcription factor. The authors find common and unique targets of PhDEF between the epidermis and mesophyll and conclude that the activity of transcription factors like PhDEF are influenced by the pre-existing environment in the cell they are expressed in. Understanding when and how a given transcription factor drives expression of unique target genes in various contexts is an important aspect of developmental biology that can be elusive outside of highly tractable model systems. As such, I think this study is of high value and has strong potential to expand our understanding of how developmental specificity is mediated by commonly employed transcriptional regulators. However, I think there are some issues with possible over-interpretation and some places where documentation of experimental design and data quality control are lacking. I elaborate on these concerns below.

Major Comments:

Line 155: Assigning mesophyll clusters by default without any positive marker genes strikes me as problematic, especially as much of the analysis rests on comparing the transcriptomes of epidermis and mesophyll cells. Can the authors perhaps leverage published scRNA-seq datasets to find potential mesophyll markers, even homologs from other species, to improve confidence in the cluster assignment?

We thank the reviewer for raising this important point. Our statement of defining mesophyll identity by default was not entirely true (and we have now removed it), since it is supported by GO-enriched terms for cluster markers. For cluster "mesophyll 3", there is a strong enrichment for photosynthesis-related genes; and for cluster "mesophyll 2", there is a strong enrichment for water transport-related genes, both functions being fulfilled by the petal mesophyll. For cluster "mesophyll 1", the most enriched GO term is "glutathione metabolic process" that rather points to stress response. These three clusters also do not express any of the epidermal-identity genes, in contrast to the clusters that we assigned as epidermal. Cluster markers from the mesophyll display the lowest enrichment of all clusters (the best cluster markers have a log2FC between 2.8 and 4.9, in contrast to a log2FC between 8.1 and 9.9 for all other clusters), which is in line with our finding that the mesophyll expresses less specific genes than the epidermis, and suggests a basal identity with a transcriptomic signature that is less clear than in the epidermis. Therefore, it is not entirely trivial to find positive marker genes for the mesophyll with a strong specificity.

In order to be more transparent about the expression patterns of the genes we selected to assign cluster identity, we modified Figure S3 to include DotPlots of selected photosynthesis-related genes, histone genes, S-phase genes and vasculature genes in the same figure, to compare with DotPlots of epidermal genes and pigmentation genes from Figure 1D, to allow for an informed comparison. Our conclusions remain the same as previously: epidermal clusters are defined based on the specific expression of epidermal genes and/or pigmentation genes. We notice, however, that the cluster "upper limb epidermis" strongly expresses photosynthesis genes, which is likely why it is close in the UMAP space to the "mesophyll 3" cluster. We have no explanation for that, but the extremely high expression of pigmentation genes in this cluster, however, identifies it as epidermal without a doubt. We have also added in Figure S3 the DotPlots of expression levels of homologs of 15 epidermis-enriched and 9 mesophyll-enriched genes from tobacco petal scRNA-Seq published by Kang et al. (doi: 10.1111/nph.17992). This shows that our definition of epidermal and mesophyll clusters and the one from Kang et al. generally overlap.

Line 228: Through the description and interpretation of the ChIP-seq dataset, the authors use the fact that peaks are more abundant and bigger in the epidermis to conclude that binding of PhDEF is "stronger" in the epidermis. This implies a difference in physical interaction between the TF and the DNA that I don't think can be concluded from the data presented. This could be confounded by biology; if expression of PhDEF is more heterogeneous in the mesophyll than in the epidermis, the peaks from that tissue will be averaged out and appear smaller when in fact the binding is the same strength. This could also be a technical artifact if the ChIP was less efficient in one sample versus another. This conclusion requires reinterpretation. The authors have the power to address this at least partially with the scRNA-seq by measuring PhDEF heterogeneity. I believe assessing ChIP efficiency would have required a spike in control, but perhaps there is a computational way to address this. It is important to discuss these confounding factors in the text.

This is indeed another important point. We agree that the word "stronger", to describe PhDEF binding in the epidermis, was not appropriate and we have replaced it with "more frequent" which is a more factual interpretation of our results. We also agree that even this interpretation depends on potential ChIP artifacts that we have now evaluated.

We have used our WT scRNA-Seq data to explore the heterogeneity of PhDEF expression in the mesophyll and the epidermis, as suggested. The barplot in Figure S10B represents the number of cells (y-axis) with given PhDEF RNA counts (x-axis) in the clusters that we assigned as epidermis (left) and mesophyll (right). We have performed this analysis after removing cells that do not express PhDEF at all, which represents 47.7% and 47.4% of epidermal and mesophyll cells, respectively, hence very similar proportions. The distributions of expression of PhDEF in the epidermis and in the mesophyll are within the same ranges, with a slightly higher expression of PhDEF in the mesophyll than in the epidermis. The coefficients of variation (cv) of the two distributions are similar and slightly higher in the epidermis (cv = 0.34 in the mesophyll and cv = 0.36 in the epidermis, p = 0.00106 with Feltz and Miller’s asymptotic test). Therefore, it appears that the expression of PhDEF is actually higher and slightly less variable in the mesophyll than in the epidermis, meaning that it should not result in averaging out the peaks detected. This relies on the assumption that PhDEF protein levels are directly correlated with PhDEF RNA levels, which we have not explored in this study and remains a limitation. We have included this analysis lines 275-278 and Figure S10B.

Regarding ChIP efficiency, we had run different tests prior to sequencing: first, we have tested different amounts of chromatin, keeping the quantity of antibody unchanged, and tested ChIP enrichment by qPCR on a set of two positive (PhDEF and Pos2) and one negative (Neg1) control binding sites. Second, after selecting the best chromatin quantity, we have performed 4 independent ChIP replicates for each genotype (split between two assays named ChIP-1 and ChIP-2) and measured ChIP efficiency by qPCR. This is depicted in Figure S10A, with the replicates chosen for sequencing highlighted with an orange star.

We have now explained in greater detail in the Methods our preliminary tests. There is indeed variation of enrichment between replicates, and particularly between assays here (ChIP-1 vs. ChIP-2), which is inherent to the ChIP experiment. It might be particularly prominent in our case due to our custom antibody directed against PhDEF, in contrast to commercial antibodies that are commonly used in ChIP experiments with tagged transgenic lines. However, we see consistently lower enrichment for star as compared to wico and WT, in line with the more frequent epidermal binding of PhDEF. We have followed the ENCODE guidelines for our analysis pipeline, in particular applying the IDR. We have now added other mapping statistics in Table S5 including the FRiP (fraction of reads in peaks) metric, that is in the range of expected values but is lower for star (around 0.5%) than for WT and wico (0.7-1.2 %), again consistent with the more frequent epidermal binding of PhDEF.

Line 376: The authors risk overinterpreting a lack of differential gene expression detection in their analysis of PhDEF binding profiles. This can be affected by how deeply a library was sequenced or how many cells were analyzed per cell type. A gene might not be found to be DE if low depth or few cells resulted in noise or dropout. Lack of detection does not mean lack of differential regulation so the biological relevance of this portion of the analysis should be interpreted with caution.

We have added to this new version of the manuscript an intersection between ChIP-Seq and bulk RNA-Seq in WT, star and wico, as bulk RNA-Seq is much more sensitive than scRNA-Seq in detecting lowly expressed genes. We have also added the sentence that bulk RNA-Seq "better captures lowly expressed genes" than scRNA-Seq, line 333. This intersection revealed an association between epidermal DEGs (star-specific DEGs) and the presence of epidermal+shared binding sites for PhDEF. We have modified our conclusions accordingly.

Line 476: The authors state there is a mismatch in developmental timing between the RNAseq and ChIP datasets. Why is this? This is mentioned briefly in the Discussion, but has the potential to be majorly confounding to the joint interpretation of the ChIP and RNAseq datasets. This experimental design choice should be justified more thoroughly and a consideration of the limitations it brings to data interpretation should be more prominent in the text.

This concern has also been raised by the first reviewer, and we have now added to our study an intersection between ChIP-Seq and bulk RNA-Seq performed at the same stage. We have also explained the technical reasons for performing scRNA-Seq at a mature stage only (lines 104-107). Indeed, the intersection between bulk RNA-Seq and ChIP-Seq performed at the same stage revealed a significant association between epidermal DEGs (star-specific DEGs) and the presence of epidermal+shared binding sites for PhDEF. Testing for individiual binding categories, we could also detect an enrichment of epidermal+shared binding sites for epidermal-specific DEGs identified by scRNA-Seq. Therefore, we have now stated that “layer-specific binding and transcriptional regulation are partially linked, at least in the epidermis” (line 354).

Minor Comments:

Line 229: The authors compare correlations between pseudo-bulked transcriptomes and argue that in the star mutant the epidermis adopts a mesophyll-like identity. The correlation between epidermis and mesophyll in star is 0.97 and the correlations were 0.94 and 0.93 in the other genotypes tested. What is the meaningful cutoff for saying the transcriptomes are similar or not? Is 0.97 so much higher than 0.94 that this conclusion is supported?

The comparison of pseudo-bulk transcriptomes is a very global and exploratory approach. Given the high number of genes underlying these pseudo-bulk datasets, any difference in the correlation between them is statistically significant, which is not very informative. We agree with this reviewer that the interpretation of these correlation coefficients is somewhat arbitrary. We have simplified this part of the manuscript and have mostly focused on comparing star and wico pseudo-bulk transcriptomes to the WT ones, but not to each other's, which aligns well with our main message of a specific epidermal identity, easily shifting to a mesophyll-identity when PhDEF is missing or not entirely functional. We have also removed Figure 2E to a supplementary figure to give less emphasis to this analysis.

Line 264: What are the "manually chosen thresholds for differential expression"? Can the authors explain and justify this? There is very little detail on this in the materials and methods and this raises some concerns regarding how a threshold was chosen.

Seurat gives a default threshold of 0.25 for log2FC, which we found to be very permissive. In order to capture the most informative targets of PhDEF, but still to capture a meaningful number of targets, we empirically decided to increase this threshold to 0.75. On the WT scRNA-Seq dataset, we observed that the layer-specificity factor was also capturing meaningful differences in layer-specific expression (see Figure 1E). We chose a cut-off at 10% since it was the lowest to give a significant difference in the numbers of epidermis- vs. mesophyll-enriched genes in the WT petal. We have added these explanations in the methods.

Figure 3G: Could this plot be annotated with the classification of peak layer specificity? It is a little difficult for me as the reader to keep up with all the categories in the text, and showing them in the figure might make that easier to follow.

We have now annotated the Venn diagram in Figure 3G with the classifications of binding profiles.

Figure 4A: A comparison of only two cell categories should not use scaled expression, as this can over-emphasize small differences in gene expression. Can this be replaced with a dot plot that uses unscaled expression values?

We thank the reviewer for noticing this issue, we have built a DotPlot with unscaled values and replaced it in Figure 4A, which does not change our conclusions. We have also used unscaled values in Figure S14.

Figure 4C: Could this be represented more legibly with stacked, space-filled bar charts? As is, this is difficult to read, and might be impossible for someone who is color blind. In addition, the authors claim this analysis shows similar proportions across all categories, but I wonder if that would hold true if they performed an over-representation analysis normalized to the categories shown in "all genes expressed". This could allow them to statistically test whether there are real differences in representation among the categories.

We have now used a different and color-blind-friendly palette for pie charts of PhDEF binding profiles. Following reviewers' comments, we have analyzed the intersection of bulk RNA-Seq with ChIP-Seq (both performed at the same stage), and performed Chi2 goodness-of-fit tests that indeed support some association between binding profile and regulation profile, although this remains limited.

Supplemental Figure 2: It's great that the authors include these metrics, but it would be ideal to also include the plots of standard QC metrics for scRNA-seq such as those found here to give a better sense of per cell quality: https://satijalab.org/seurat/articles/pbmc3k_tutorial

In addition, it is important to include QC metrics for ChIPseq, which I did not find in the supplement. Metrics such as FRiP are important for interpreting ChIP library quality.

We have now added the standard QC plots (Feature number per cell and RNA counts per cell) to Figure S2, as suggested. Mitochondrial and ribosomal genes are not annotated in the Petunia axillaris nuclear genome that we used, therefore we could not compute mitochondrial or ribosomal gene counts. We have removed cells expressing less than 200 genes, but did not apply any upper thresholds as there were no obvious outliers.

FastQC reports for scRNA-Seq and ChIP-Seq have been deposited at here, as indicated in the Methods.

We have also added ChIP metrics in Table S5, including the number of reads, duplicated reads, mapped reads and computed the FriP score. This score ranges between 0.5 % and 1.2 %, which is satisfactory and above the minimum recommended score by ENCODE of 0.3 %.

Line 654: What model was used for DESeq2?

We have used default parameters for DESeq2, ie a negative binomial GLM fitting and Wald significance tests. We have added this information in the Methods.

Line 752: Can the authors justify why peaks were called separately on input and ChIP samples rather than allowing MACS2 to call peaks in the ChIP sample over input background? That differs from the standard MACS2 pipeline and no explanation for this is provided in the text.

Our analysis pipeline has indeed been customized, in particular to detect peaks in our positive control PhDEF, for which a binding site of PhDEF in the promoter has been demonstrated experimentally by others in many different species. This binding has a strong experimental support, and we expected PhDEF to bind to its own promoter in the two cell layers. Our ChIP-Seq results show a posteriori that this particular peak is far from being the strongest one over the genome; therefore, we believe it represents a good control to detect binding enrichment for average targets. We have first tried the standard MACS2 pipeline that calculates the enrichment of IP over Input, but we could only detect PhDEF binding for one WT and one wico replicate, although the peaks were visually clear in the two replicates. Our input sample being generally noisy, we explored how separate peak calling between IP and Input would behave (as already done in e.g. Durand et al., 2023, doi: 10.1093/plcell/koad025). We also explored how thresholds for FDR in MACS2, and IDR thresholds for reproducibility between IP samples, would influence peak detection in IP and Input.

We found that calling peaks on the IP with a relaxed FDR (0.1), then applying the IDR at 0.1, allowed the capture of PhDEF binding to its own promoter in the two wico replicates (but still not in WT, because the peaks were lost after applying the IDR threshold). No peak was detected in the input with these settings, however for other genes we observed spurious peak detection in the input, therefore we decided to lower the FDR thresholds for input peaks to 0.05. Our choices have been made in an attempt to increase specificity at the risk of losing sensibility, and we probably lose true binding events. Considering that this ChIP has been performed on the endogenous PhDEF protein directly, and in chimeric flowers that only express PhDEF in half of the tissue, adapting the ChIP analysis pipeline was a necessary step.

We have now added a few lines in the Methods (lines 696-706) to explain our rationale.

**Reviewer #2 (Significance):**

General Assessment:

Strengths: The authors employ a unique and powerful mutant system to explore a fundamental developmental biology question. In addition, the datasets generated will likely be useful to other researchers working in petunia or flower development.

Limitations: While the mutant system employed here is a creative way to get at tissue-layer specific transcription factor activity, the ChIP samples still include heterogeneous cell types, which may impact the findings presented here.

Advance: This study uses a unique system to test the function of a transcription factor in distinct cell types. As stated above, understanding when and how a given transcription factor drives expression of unique target genes in various contexts is an important aspect of developmental biology.

Audience: I believe this work will be of primary interest to the plant development, single cell, and chromatin biology communities. These are specialized, basic research communities.

Reviewer Expertise: I am a plant developmental biologist who works with multiple modes of cell-type-specific NGS datasets including bulk and single cell RNAseq and ChIPseq among others.

**Reviewer #3 (Evidence, reproducibility and clarity):**

Summary:

This study addresses a fundamental but underexplored aspect of homeotic gene function: how regulators of cell identity act during late stages of organ development. The authors take advantage of layer-specific mutants of the MADS-box gene PhDEF in Petunia hybrida to dissect the roles of this floral identity regulator in the epidermis and mesophyll. By combining single-cell RNA sequencing with ChIP-seq analyses in wild-type and mutant chimeric petals, the work demonstrates that, although PhDEF is expressed at comparable levels in both petal layers, it binds to and regulates a substantially larger and more layer-enriched set of genes in the epidermis than in the mesophyll. The identification of both layer-specific and shared PhDEF binding sites supports a model in which pre-existing layer identity modulates the regulatory output of homeotic transcription factors.

Major comments:

Dissecting PhDEF binding preferences in the epidermis versus the mesophyll using the star and wico mutants is a clever and powerful approach. However, conclusions involving cell identity should be drawn with caution for two reasons. First, cell identities appear to be altered in the mutants: scRNA-seq data suggest that even cells assigned to the same cluster can be molecularly distinct across genotypes. For example, the transcriptomes of wild-type epidermis and mesophyll are highly similar (Pearson correlation R = 0.94), yet both show lower correlation with epidermal cells from star or wico petals. These results raise questions such as are the cells identified as epidermal cells really strictly epidermal in the mutants? Do you need to take into consideration cell composition of the mutants when you do differential expression analysis?Second, PhDEF is expressed in both epidermal and mesophyll clusters in all genotypes, albeit at different levels and in fewer cells in the mutants. As a result, the ChIP-seq profiles should be interpreted as cell type-enriched rather than cell type-specific.

We fully agree with this reviewer's comments, and alterations in cell identity in the star and wico mutants is indeed a main pitfall for our analysis. The phdef mutation alters the transcriptomic signatures of cells physically located in the epidermis or in the mesophyll, which can result in their artificial clustering with cells located elsewhere in the petal. It might be particularly true for the epidermis, since we see strong depletion in epidermal clusters in the star flowers (and even in the wico flowers), whereas the mesophyll is not much affected in wico flowers. As a result, it is likely that we lose many epidermal cells in star flowers that end up labeled as mesophyll cells, resulting in the under-estimation of DEGs in this layer. We had explored other possible ways to define epidermal or mesophyll cells in our dataset, based for instance on PhDEF or PhGLO1 expression, but since for the majority of cells PhDEF expression is simply not captured, we would have wrongly assigned phdef mutant identity to WT cells. In spite of this limitation, we find more DEGs in the epidermis than in the mesophyll, showing that even an underestimation of DEGs in the epidermis does not affect our main conclusion, which is that PhDEF has a major regulatory action in the epidermis. We have explicitly written this limitation in our manuscript, lines 234-236.

We agree that ChIP-Seq profiles are rather cell type-enriched than cell type-specific, which we have stated explicitly in the sentence line 308, saying that "differential binding between layers is quantitative". However, for simplicity we prefer to retain the term "specific" since our conclusions are based on the definition of peaks that can either be present or absent in a given genotype, and hence in a given cell layer.

And there are a few things that need clarification:

a) Protoplasting and tissue dissection analyses suggest that mesophyll cells constitute more than 80% of the cells in wild-type petals, whereas the scRNA-seq data indicate a substantially lower proportion. Could this discrepancy reflect technical biases in cell recovery or capture efficiency, or issues related to cell identity assignment during clustering and annotation? Notably, the scRNA-seq data from star and wico petals show mesophyll proportions close to 80%. Is this difference due to an increased abundance of mesophyll cells in the mutants, or could it instead reflect differences in transcriptomic separability? In wild-type petals, the epidermal and mesophyll transcriptomes are highly correlated and express similar numbers of genes, with epidermal cells distinguished mainly by higher expression of a subset of genes. This raises the possibility that mesophyll cells in the wild type occupy a more plastic or less differentiated transcriptional state and may therefore be misclassified as epidermal cells, whereas disruption of regulatory mechanisms in the mutants enhances transcriptional divergence and alters cell clustering outcomes.

Our protoplasting and tissue sections show that the mesophyll should represent 80% of cells in WT tissue, while we estimate it at 70% in our WT scRNA-Seq data based on our cluster assignment (mesophyll = 60% + vasculature = 10%, that we separated from the mesophyll cells but is actually embedded within this tissue). This is not a very strong difference, and considering the multiple steps that protoplasting and cell capture entail, we considered that this was reasonably close to the expected proportions.

In the star and wico flowers, we assign mesophyll identity to a greater proportion of cells, but as explained above, we believe that cells with altered epidermal identity are easily clustered as mesophyll cells, since they lose their specific transcriptomic signature. This is actually in line with one of the main messages of our article, that petal epidermis transcriptional identity is highly specific.

b) Cluster 0 appears to show internal heterogeneity, as the expression patterns of KCS3 and LLE2 are largely mutually exclusive. Do these patterns reflect the presence of distinct epidermal cell types within the limb that are currently grouped into a single cluster?

Indeed, there appears to be some internal heterogeneity within cluster 0. Since our main focus was to compare epidermal and mesophyll clusters, we did not explore further the heterogeneity within epidermal clusters and kept a coarse resolution.

c) Clusters 0 and 7 both exhibit high expression of pigmentation genes, while cluster 7 additionally shows strong enrichment for cell division genes. Are cell cycle genes the primary features distinguishing these two clusters? If cell cycle effects are regressed out, would cluster 7 merge with cluster 0, potentially yielding a more continuous cell state trajectory and helping to resolve the pattern noted in point (b)?

To answer one of Reviewer 1's comments, we have added additional DotPlots to better describe our clusters, in Figure S3. Clusters 0 (limb epidermis), 6 (upper limb epidermis) and 7 (replicating cells) exhibit high expression of pigmentation genes (in particular cluster 6), as depicted in Figure 1D. Cluster 7, consisting of only 30 cells, is the only cluster expressing histone genes and S-phase genes. It is possible that these few cells are cluster-6 cells that are replicating, but considering the very low number of cells involved, we have not explored any further their identity and decided to remove them, as it would only marginally affect the conclusions of our analyses. It is indeed surprising that cluster 6 (upper limb epidermis) is quite distinct in the UMAP space to the other epidermal clusters 0 (limb epidermis) and 4 (upper tube epidermis), and we have not observed similar situations in other scRNA-Seq studies. We speculate that this is due to the joint expression of anthocyanin-related and photosynthesis-related genes, which convey a very strong transcriptomic signature to these cells that distinguish them from other epidermal cells.

d) Pearson correlation is largely driven by highly expressed genes and may therefore be insensitive to changes in cell identity markers. The conclusions that the less clear separation of mesophyll and epidermal cells in star is due to altered cell identity would be more convincing if supported by independent validation, such as in situ hybridization or reporter analyses, to directly visualize molecular alterations in the relevant cell types when comparing wild-type and mutant tissues.

Following another reviewer's comments, we have now given less emphasis on the Pearson correlation analysis, that was to some extent subjective. Therefore, our conclusion that mesophyll and epidermal cells in star are less separated than in WT has been removed.

Minor comments:

(1) For color-coded figure legends (e.g., Fig. 1C), please also include the cluster numbers. This would facilitate interpretation, particularly for readers with reduced color sensitivity.

We have now used colour-blind-friendly palettes and we have added cluster numbers in Figure 1C.

(2) For figures containing abbreviations (e.g., Fig. 1D, st. / ca.), please explicitly define all abbreviations in the figure legend.

We have defined all abbreviations in the figure legends.

(3) For all UMAP figures, and for figures involving comparisons across clusters, please use consistent color schemes for the same clusters throughout the manuscript.

We have modified color schemes across the manuscript for colour-blind-friendly palettes, consistently used throughout the manuscript.

(4) In Fig. S6, the plot showing all genes does not exactly match Fig. 1C, although it appears to represent the same data. Please use the same version of packages, seed values and parameters for all UMAP plots to avoid such discrepancies.

Figure S6 is the result of integrating with Harmony the WT dataset (as shown in Figure 1C) with WT datasets after removing genes differentially expressed by the protoplasting process. Therefore these UMAPs are a result of integrating different datasets than in Figure 1C, which changes the shape of the UMAPs but cannot be controlled by the seed values, to our knowledge.

**Reviewer #3 (Significance):**

Overall, this work significantly advances our understanding of late homeotic gene function. It establishes compelling evidence for how developmental context constrains transcription factor activity and offers broadly relevant insights for studies of organ patterning. The combination of genetic mosaics with single-cell and chromatin-level analyses represents a powerful and generalizable strategy that will be of interest to both plant developmental biologists and researchers studying transcriptional regulation.

My expertise: single cell genomics, chromatin biology and plant development.
